# Longitudinal Evolution of Radiomic Features in Radiation-Induced Necrosis During Follow-Up of Brain Metastases: A Pilot Study

**DOI:** 10.3390/diagnostics16152339

**Published:** 2026-07-26

**Authors:** Claudia Tocilă-Mătășel, Sorin Marian Dudea, Gheorghe Iana

**Affiliations:** 1 Department of Radiology, Faculty of Medicine, Iuliu Hatieganu University of Medicine and Pharmacy, 400012 Cluj-Napoca, Romania; 2 Medima Health SA, 060254 Bucharest, Romania

**Keywords:** radiomics, feature stability, longitudinal analysis, MRI

## Abstract

**Background:** Radiomic analysis enables quantitative characterization of post-radiotherapy brain lesions beyond conventional imaging. However, the longitudinal behavior of radiomic features, particularly across different MRI scanners, remains insufficiently explored. This pilot study aimed to explore the longitudinal evolution of radiomic features in radiation-induced necrosis following radiotherapy for brain metastases by comparing trajectories obtained from MRI follow-up performed on the same scanner with follow-up acquired across different scanners. **Methods:** This retrospective pilot study included 40 radiation necrosis lesions divided into a same-scanner cohort (20 lesions) and a mixed-scanner cohort (20 lesions). Each lesion underwent three sequential post-contrast T1-weighted MRI examinations, resulting in 120 longitudinal lesion-level observations. Images underwent standardized preprocessing, three-dimensional lesion segmentation, and radiomic feature extraction. Feature robustness to segmentation variability was assessed using the coefficient of variation and intraclass correlation coefficient, and only robust features were retained. Longitudinal coherence was evaluated using the Δ-feature metric quantifying longitudinal variability. **Results:** Seventy-six of 107 radiomic features (71%) were robust to segmentation perturbations. Substantial longitudinal variability was observed even in the same-scanner cohort. Shape features demonstrated lower longitudinal variability, whereas texture-based features showed descriptively higher variability in the mixed-scanner cohort. However, these differences were not statistically significant after false discovery rate correction. **Conclusions:** This pilot study highlights the importance of validating longitudinal radiomic feature stability before interpreting temporal radiomic changes as biological phenomena or incorporating radiomic biomarkers into clinical decision-support models.

## 1. Introduction

Brain metastases are frequently treated with stereotactic radiotherapy or radiosurgery, leading to improved local control and prolonged survival. However, radiotherapy may be complicated by radiation-induced necrosis, which can closely mimic tumor progression on conventional magnetic resonance imaging (MRI) [1]. Differentiating between these entities is critical, as management strategies differ substantially [2,3].

Artificial intelligence (AI) has emerged as a rapidly evolving field in medical imaging, offering computational approaches that support diagnosis, prognosis, treatment planning, and prediction of therapeutic response. AI encompasses several methodological approaches, including conventional machine learning and deep learning, which differ in their computational architecture, data requirements, interpretability, and clinical applications. In neuroimaging and neuro-oncology, these methods have been increasingly investigated for lesion detection, disease characterization, outcome prediction, and clinical decision support, reflecting the growing role of AI in quantitative image analysis and precision medicine [4,5,6].

Radiomics represents one of the main AI-assisted quantitative imaging methodologies. It converts routinely acquired medical images into high-dimensional quantitative features describing lesion shape, intensity distribution, and textural heterogeneity. These handcrafted imaging features can subsequently be incorporated into machine learning algorithms to develop diagnostic, prognostic, and predictive models, providing an interpretable bridge between conventional medical imaging and artificial intelligence. Several studies have demonstrated promising performance of radiomic models in distinguishing radiation necrosis from recurrent tumor, often achieving high diagnostic accuracy [7]. Despite promising results from high-performing single-center radiomics models and increasing multicenter initiatives, the effect of scanner and center variability on longitudinal radiomic feature stability remains insufficiently explored [8,9]. Importantly, several studies have shown that radiomic features exhibit substantial intrinsic sensitivity, with limited repeatability and reproducibility even under consistent imaging conditions [10]. Consequently, longitudinal feature stability represents an essential prerequisite before radiomic biomarkers can be reliably interpreted as reflecting biological evolution or incorporated into AI-driven predictive models.

In routine oncology workflows, patients frequently undergo longitudinal MRI follow-up across different institutions and scanners, either due to referral patterns, geographic relocation, or healthcare system constraints. Changes in scanner hardware, acquisition parameters, and reconstruction algorithms can introduce variability in radiomic features that is unrelated to true biological change [11].

This pilot study aimed to explore the longitudinal behavior of radiomic features in radiation-induced necrosis following radiotherapy for brain metastases by comparing longitudinal trajectories between same-scanner and mixed-scanner follow-up cohorts. 

## 2. Materials and Methods

### 2.1. Study Design and Population

This retrospective pilot study was conducted at a single imaging center. Cases were identified through a retrospective search of the institutional PACS database and included patients who were previously treated with radiotherapy for brain metastases and were later diagnosed with radiation-induced necrosis based on imaging findings and clinical follow-up. Only lesions with three sequential MRI examinations available for longitudinal analysis were included.

External examinations were performed at multiple external imaging centers using heterogeneous 1.5 T and 3 T MRI systems and acquisition protocols. These external studies were provided by the patients for comparative clinical interpretation and were subsequently retrieved from the institutional PACS archive. Although detailed acquisition parameters (e.g., TR/TE or contrast timing) varied, all external examinations included at least one high-resolution post-contrast T1-weighted sequence with a slice thickness ≤ 1.5 mm. 

All MRI examinations performed at our center were acquired on a 3 T MRI system (GE Signa Pioneer, GE Healthcare, Milwaukee, WI, USA) equipped with a 21-channel phased-array head and neck coil. The standard post-contrast sequence used for lesion assessment was a 3D T1 FSPGR acquisition with 1 mm isotropic voxels and no interslice gap.

Radiation necrosis was diagnosed based on imaging findings, including irregular enhancement, central necrotic components, low relative cerebral blood volume (rCBV) when available, and longitudinal imaging behavior not consistent with progressive tumor growth, in conjunction with clinical assessment by a multidisciplinary neuro-oncology team. Histopathological confirmation was not available for the included cases, reflecting routine clinical practice.

Radiomic analysis was performed on post-contrast T1-weighted images, which were available for all examinations and constituted the reference sequence for longitudinal assessment.

Two analytical cohorts were defined based on scanner continuity during follow-up:

(a) Same-scanner cohort (n = 20 lesions), in which all three MRI examinations were acquired on the same scanner.
(b) Mixed-scanner cohort (n = 20 lesions), in which MRI examinations were acquired on different scanners during follow-up.

### 2.2. Image Preprocessing

All MRI examinations underwent an identical standardized preprocessing pipeline prior to radiomic feature extraction in order to minimize acquisition-related variability. All preprocessing was performed using Python (version 3.11, 64-bit) [12]. The following steps were applied consistently across all scans.

#### 2.2.1. Bias-Field Correction

Low-frequency intensity inhomogeneity was corrected using N4ITK bias-field correction to improve intensity consistency across the brain scan.

#### 2.2.2. Spatial Resampling

All images were resampled to an isotropic 1 × 1 × 1 mm^3^ voxel grid using third-order spline interpolation. This ensured uniform voxel geometry across patients and scanners, meeting IBSI recommendations for radiomic reproducibility.

#### 2.2.3. Intensity Normalization

For each scan, intensities were normalized using a Z-score standardization. The reference mean and standard deviation were derived from a standardized region-of-interest (ROI) placed within contralateral normal-appearing cerebral white matter to reduce inter-scan intensity variability.

### 2.3. Lesion Segmentation

Lesions were segmented using a semi-automatic three-dimensional region approach in 3D Slicer (version 5.8.1; Surgical Planning Laboratory, Harvard Medical School, Boston, MA, USA) [13], followed by manual refinement by a radiologist with experience in neuro-oncology imaging. Segmentation was performed on the entire lesion volume, including all contrast-enhancing and non-enhancing components, while excluding surrounding edema. Each time point was segmented independently on its corresponding post-contrast T1-weighted scan.

### 2.4. Radiomic Feature Extraction

Radiomic features were extracted using 3D Slicer, using the Slicer Radiomics extension (PyRadiomics library; version 8426cdf), yielding 107 features per lesion across the following classes: shape/geometry, first-order statistics, GLCM, GLRLM, GLSZM, GLDM, and NGTDM texture features. 

Following image preprocessing, a fixed bin width of 0.24 was applied consistently to all examinations to ensure a common gray-level discretization scale throughout the longitudinal analysis. This resulted in a comparable number of occupied gray levels across the two cohorts (same-scanner: median 162, IQR 101.3–196.8; mixed-scanner: median 168, IQR 120.8–205.5).

### 2.5. Segmentation Robustness Analysis

Segmentation robustness was assessed only in cases acquired on the same scanner to avoid confounding effects related to inter-scanner variability.

Lesion ROIs ranged from approximately 113 to 20,425 voxels (median 1078.5).

To assess segmentation robustness, each lesion underwent circumferential morphological dilation and erosion of ±1 voxel, applied uniformly to the lesion boundary to simulate small variations that may occur during manual segmentation. These perturbed segmentations were used exclusively for robustness analysis. Although this represents a minor geometric perturbation, radiomic features, particularly texture and surface-related features, may remain sensitive to small boundary changes.

For each radiomic feature, robustness was quantified using two complementary metrics: the coefficient of variation (CV) and the intraclass correlation coefficient (ICC).

The coefficient of variation (CV) was calculated across the three segmentation variants (original, dilated, eroded) and expressed as a percentage, reflecting the relative variability of feature values induced by minor segmentation changes.

The intraclass correlation coefficient ICC(2,1) was computed to assess absolute agreement among segmentation variants, treating the three masks as independent raters within a two-way random-effects model.

Based on the combined performance of CV and ICC, radiomic features were classified into three robustness categories:

- Excellent robustness: ICC(2,1) ≥ 0.90 and median CV ≤ 10%;
- Good robustness: ICC(2,1) ≥ 0.75 and median CV ≤ 20%;
- Sensitive: features not meeting the above criteria.

For each radiomic feature class, the proportion of segmentation-robust features was calculated as the percentage of features classified as excellent or good relative to the total number of features in that class:

$$\mathrm{Robust}\;\mathrm{percentage}=\frac{N_{\mathrm{excellent}}+N_{\mathrm{good}}}{N_{\mathrm{total}\text{\_}\mathrm{class}}}\times 100.$$

Only features classified as segmentation-robust (excellent or good) were retained for subsequent longitudinal stability and scanner variability analyses.

### 2.6. Δ-Feature-Based Longitudinal Variability Magnitude

Δ_feature was computed separately for the same-scanner and mixed-scanner cohorts.

For each lesion and each segmentation-robust radiomic feature, absolute pairwise differences between consecutive time points were computed:

$$\Delta_{21}=|y_{T2}-y_{T1}|,\Delta_{32}=|y_{T3}-y_{T2}|$$

The maximum absolute change across follow-ups was defined as:

$$\Delta_{\max}=max(\Delta_{21},\Delta_{32})$$

At the feature level, longitudinal instability was summarized as:

$$\Delta_{\mathrm{feature}}=\mathrm{median}(\Delta_{\max})$$

Feature-level summaries were subsequently aggregated by radiomic feature class to facilitate class-level comparisons. 

Differences in longitudinal variability (Δ_feature), defined as the maximum absolute change between consecutive time points (max (|T2 − T1|, |T3 − T2|)), were assessed between the same-scanner and mixed-scanner cohorts using the Mann–Whitney U test, given the non-normal distribution and small sample size. To account for multiple comparisons across radiomic features, *p*-values were adjusted using the Benjamini–Hochberg false discovery rate (FDR) procedure. A corrected q-value < 0.05 was considered statistically significant.

## 3. Results

### 3.1. Patient and Lesion Characteristics

A total of 40 lesions from 33 patients with post-radiotherapy radiation necrosis were included in this pilot analysis. Each lesion was evaluated on three longitudinal post-contrast T1-weighted MRI examinations acquired at different follow-up time points, resulting in a total of 120 lesion-level observations. Baseline demographic, treatment, and lesion characteristics of the same-scanner and mixed-scanner cohorts are presented in Table 1. Panel A summarizes patient-level demographic characteristics, whereas Panel B summarizes le-sion-level treatment characteristics, lesion volumes, and follow-up duration.

**Figure 1:**
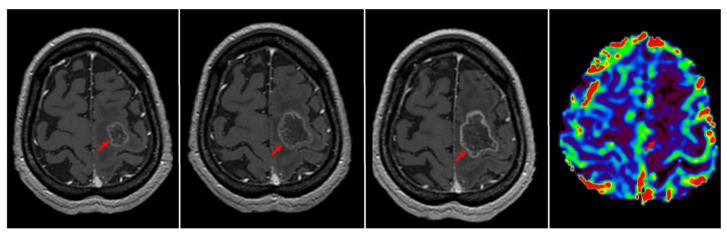
Axial post-contrast T1-weighted MRI of a patient with malignant melanoma brain metastasis treated with stereotactic radiosurgery, acquired at three follow-up time points (19, 23, and 25 months after treatment), showing an irregularly enhancing lesion with progressive dimensional enlargement and central necrotic components (arrows). The relative cerebral blood volume (rCBV) map from the final follow-up examination demonstrates no focal areas of increased perfusion within the lesion, supporting the diagnosis of radiation-induced necrosis despite interval size enlargement.

**Figure 2:**
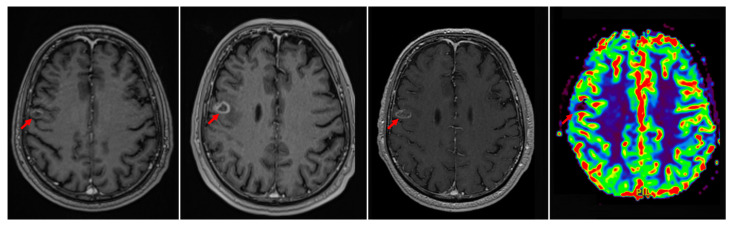
Longitudinal axial post-contrast T1-weighted MRI follow-up of a patient with a lung cancer brain metastasis treated with single-fraction stereotactic radiosurgery, with examinations acquired on different MRI scanners at 14, 18, and 24 months after treatment. Serial imaging demonstrates progressive dimensional enlargement of the treated lesion. The relative cerebral blood volume (rCBV) map obtained at the final follow-up shows no focal areas of increased perfusion, supporting radiation-induced changes rather than tumor recurrence despite interval lesion enlargement.

Representative examples of radiation-induced necrosis from the same-scanner and mixed-scanner cohorts are presented in Figure 1 and Figure 2, respectively.

### 3.2. Segmentation Robustness of Radiomic Features

Of the 107 extracted radiomic features, 76 (71.0%) met the predefined segmentation robustness criteria and were retained for subsequent longitudinal analysis.

Table 2 summarizes the distribution of segmentation-robust radiomic features across feature classes. 

Among shape features, diameter- and axis-based metrics demonstrated good robustness, whereas volume- and surface-related descriptors were more sensitive to boundary perturbations.

First-order (83%) and GLCM (79%) features demonstrated the highest proportion of segmentation-robust features, whereas GLRLM, GLSZM, and GLDM features showed intermediate robustness. NGTDM features exhibited the lowest robustness (40%). 

A summary of segmentation robustness metrics by feature class is provided in Table 3. Robust feature classes were characterized by low median CV values and high median ICC(2,1).

Among segmentation-robust features, shape and texture features from the GLCM, GLRLM, GLSZM, and GLDM classes demonstrated low variability and high agreement across segmentations, as reflected by low median CV values and high median ICC(2,1). In contrast, NGTDM features exhibited substantially higher variability and lower agreement across segmentations. Linear shape descriptors, including diameter- and axis-based metrics, were also among the most stable geometric features.

### 3.3. Longitudinal Variability of Radiomic Features

Longitudinal feature variability was quantified using Δ_feature, defined as the median of the maximum absolute difference observed between consecutive follow-up examinations (Δ_max).

**Figure 3:**
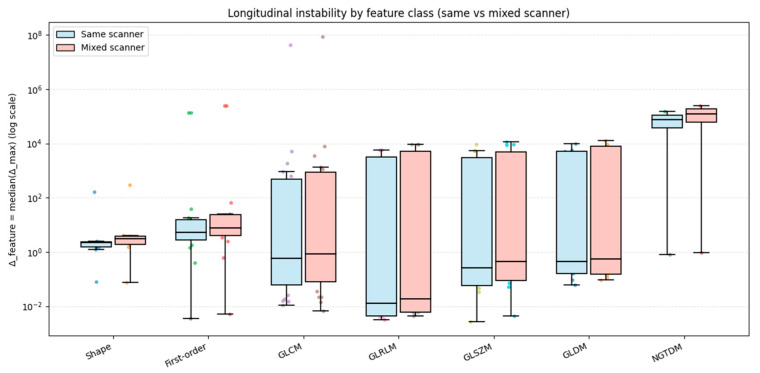
Δ_feature across radiomic feature classes for same-scanner and mixed-scanner cohorts.

Figure 3 illustrates longitudinal variability across radiomic feature classes on a logarithmic scale. 

Across most feature classes, median Δ_feature values tended to be higher in the mixed-scanner cohort than in the same-scanner cohort. This is reflected by higher median Δ values in the boxplots. 

Shape features generally show lower variability, with relatively small Δ values, suggesting greater robustness. First-order features appear slightly more variable but remain comparatively stable. In contrast, texture-based features (GLCM, GLRLM, GLSZM, and GLDM) exhibit higher variability and broader distributions. NGTDM features show the highest apparent variability in this dataset. Overall, shape features appeared more longitudinally stable, whereas texture features exhibited greater longitudinal variability.

Distributions largely overlapped between same-scanner and mixed-scanner cohorts across all feature classes. The substantial intrinsic instability observed even in same-scanner acquisitions limited the ability to isolate scanner-related effects in this small cohort. 

Mann–Whitney U testing identified several features with nominally significant differences at the unadjusted level (lowest *p* = 0.0047). However, none of these differences remained statistically significant after Benjamini–Hochberg false discovery rate correction (all q > 0.05). 

## 4. Discussion

This study was designed as a pilot exploratory investigation to assess whether radiomic features exhibit sufficiently consistent longitudinal behavior to reveal reproducible temporal patterns and whether this behavior is influenced by changes in MRI acquisition conditions. 

From a clinical perspective, it may appear self-evident that the imaging appearance of radiation necrosis evolves over time. However, radiomics aims to quantify imaging characteristics beyond visual assessment and is increasingly proposed for longitudinal monitoring and predictive modeling. Before such quantitative biomarkers can be interpreted as reflecting biological evolution, it is necessary to understand their intrinsic temporal behavior and their susceptibility to non-biological sources of variation, including scanner-related heterogeneity. The present study was therefore conceived as a methodological exploration of radiomic stability rather than as a demonstration of clinically observable lesion evolution. 

Consistent with prior literature, radiomic features differed in their sensitivity to segmentation. This highlights the need to assess segmentation robustness prior to longitudinal interpretation [14].

Longitudinal variability was quantified using the Δ_feature metric. Shape-derived features demonstrated comparatively lower longitudinal variability, whereas first-order features exhibited intermediate variability, and texture-based features consistently showed the highest longitudinal variability. In the present study, the Δ_feature metric was primarily used to describe longitudinal variability rather than to enable quantitative comparisons across radiomic feature classes. 

Collectively, these findings highlight substantial intrinsic longitudinal variability of radiomic features, even under consistent acquisition conditions. Descriptively greater longitudinal variability was observed in the mixed-scanner cohort across most radiomic feature classes. However, these differences did not remain statistically significant after false discovery rate correction and should therefore be interpreted as descriptive trends rather than evidence of an independent scanner effect. These observations are generally aligned with the systematic review by Traverso et al., which demonstrated that the repeatability and reproducibility of radiomic features are strongly influenced by acquisition, reconstruction, segmentation, and preprocessing factors [10]. More recently, Demircioğlu highlighted that limited reproducibility and interpretability remain among the principal obstacles preventing the clinical translation of radiomics [11]. Similarly, Obuchowicz et al. demonstrated that shortening MRI acquisition time by increasing GRAPPA acceleration progressively reduced the number of reproducible texture features, emphasizing that even isolated acquisition-related technical factors may substantially influence radiomic measurements independently of underlying biological changes [15].

An important observation from this pilot study is the substantial intrinsic variability of many radiomic features, even under same-scanner follow-up conditions. Rather than identifying robust longitudinal patterns, our findings highlight the complexity of interpreting temporal radiomic changes and suggest that apparent feature variation cannot be assumed to reflect biological evolution alone. These results encourage a cautious interpretation of longitudinal radiomic analyses and emphasize the need to better understand what information radiomic features can reliably provide in clinical follow-up, potentially as complementary rather than standalone biomarkers. 

These observations are consistent with the findings of Wennmann et al., who demonstrated that only a subset of radiomic features remained reproducible across multiple MRI scanners. Shape-derived features showed the highest reproducibility, whereas texture features, particularly GLCM-, GLRLM-, and GLSZM-derived metrics, were more susceptible to scanner-related variability. Importantly, the use of reproducible features improved the generalizability of predictive machine learning models [16].

Further studies are needed to better understand which radiomic features provide reproducible longitudinal information and how such information may complement conventional imaging assessment and clinical decision-making.

This observation is particularly relevant because texture features are frequently selected by machine learning algorithms when developing radiomic signatures. As highlighted by Vrettos et al., the performance of AI-driven radiomics critically depends on the quality, robustness, and reproducibility of the extracted features. Consequently, highly variable features may reduce the generalizability of predictive models if longitudinal stability is not considered during feature selection and model development [17].

This study has several limitations. First, its retrospective design and small sample size limit statistical power and generalizability. Second, the number of longitudinal time points per lesion was limited to three examinations, which constrains the characterization of complex temporal trajectories and may oversimplify dynamic radiomic behavior. Third, although segmentation robustness was assessed using controlled perturbations, inter-observer variability was not explicitly evaluated. Fourth, some patients contributed more than one lesion, and lesion-level analyses assumed independence between lesions without accounting for potential within-patient correlation. Fifth, the study focused exclusively on post-contrast T1-weighted imaging, and findings may not directly translate to other MRI sequences. Sixth, the absence of histopathological confirmation means that some degree of diagnostic uncertainty remains unavoidable. Finally, differences in follow-up duration and treatment composition, including the higher proportion of lesions treated with whole-brain radiotherapy in the mixed-scanner cohort, represent potential confounding factors that may have influenced the observed longitudinal variability. Because time-dependent effects were not formally controlled for in the statistical analysis, the descriptive differences observed between cohorts should not be attributed exclusively to scanner-related variability, as temporal evolution and treatment-related effects may also have contributed to the observed longitudinal variability. These limitations are consistent with the exploratory nature of this pilot study and reflect the retrospective analysis of MRI examinations as they were available in routine clinical practice. Rather than providing definitive answers, the present work highlights the complexity of longitudinal radiomic analysis and underscores the need for cautious interpretation of temporal feature changes before they are interpreted as imaging biomarkers of biological evolution or incorporated into predictive models.

## 5. Conclusions

Longitudinal variability of radiomic features in radiation necrosis appears to be influenced by intrinsic feature sensitivity, as most texture features showed substantial variability even under consistent acquisition conditions. Although descriptively greater variability was observed in the mixed-scanner cohort, these differences were not statistically significant after false discovery rate correction and should be interpreted cautiously. Shape-derived features demonstrated comparatively lower longitudinal variability across both follow-up settings.

Beyond its descriptive findings, this pilot study highlights the importance of validating longitudinal feature stability before interpreting radiomic changes as biological phenomena or incorporating them into clinical decision-support models. 

Establishing longitudinally robust radiomic features may represent an important prerequisite for the development of reliable AI-driven radiomic signatures suitable for clinical decision support.

## Figures and Tables

**Table 1:** Baseline characteristics of the study cohorts.

(A) Patient-Level Characteristics		
Characteristic	Same-Scanner Cohort (20 Lesions, 17 Patients)	Mixed-Scanner Cohort (20 Lesions, 16 Patients)
Patients, n	17	16
Female sex, n (%)	9 (53%)	9 (56%)
Male sex, n (%)	8 (47%)	7 (44%)
Age, median (range), years	58 (40–79)	65.5 (37–78)
Primary tumor—lung, n (%)	12 (71%)	13 (81%)
Primary tumor—breast, n (%)	2 (12%)	1 (6%)
Primary tumor—melanoma, n (%)	1 (6%)	0
Other primary tumors, n (%)	2 (12%)	2 (13%)
**(B) Lesion-Level Characteristics**		
**Characteristic**	**Same-Scanner Cohort (20 Lesions, 17 Patients)**	**Mixed-Scanner Cohort (20 Lesions, 16 Patients)**
Lesions, n	20	20
SRS only, n (%)	16 (80%)	12 (60%)
SRS + WBRT, n (%)	4 (20%)	8 (40%)
Lesion volume across longitudinal examinations, median (range), mm^3^	1078.5 (113–20,425)	1500 (169–9244)
Follow-up duration after radiotherapy, median (range), months	11 (2–29)	13 (2–46)

**Table 2:** Segmentation robustness of radiomic features by feature class.

Feature Class	Total Features	Excellent	Good	Sensitive	% Robust
Shape	14	4	5	5	64%
First-order	18	8	7	3	83%
GLCM	24	13	6	5	79%
GLRLM	16	10	1	5	68%
GLSZM	16	10	1	5	68%
GLDM	14	8	1	5	64%
NGTDM	5	0	2	3	40%
Total	107	53	23	31	71%

**Table 3:** Summary of segmentation robustness metrics for all 107 extracted radiomic features by feature class.

Feature Class	CV Median (Range)	ICC(2,1) Median (Range)
Shape	6.0% (2.9–33.0)	0.90 (0.41–0.96)
First-order	9.9% (0.0–96.5)	0.93 (0.67–0.99)
GLCM	5.2% (0.1–81.7)	0.96 (0.52–0.98)
GLRLM	3.5% (0.02–34.0)	0.97 (0.46–0.98)
GLSZM	5.8% (0.3–33.5)	0.96 (0.45–0.99)
GLDM	5.4% (0.4–33.9)	0.97 (0.46–0.99)
NGTDM	35.0% (10.3–37.7)	0.78 (0.50–0.97)

## Data Availability

The data presented in this study are available from the corresponding author upon reasonable request. The data are not publicly available due to patient privacy and institutional restrictions.
